# Culturable endophytic fungal assemblages from *Styrax sumatrana* and *Stryax benzoin* and their potential as antifungal, antioxidant, and alpha-glucosidase inhibitory resources

**DOI:** 10.3389/fmicb.2022.974526

**Published:** 2022-11-04

**Authors:** Deni Elfiati, Sarah Asih Faulina, Laras Murni Rahayu, Aryanto Aryanto, Rizna Triana Dewi, Henti Hendalastuti Rachmat, Maman Turjaman, Mohammad Fathi Royyani, Arida Susilowati, Asep Hidayat

**Affiliations:** ^1^Faculty of Forestry, Universitas Sumatera Utara, Medan, Indonesia; ^2^Research Center for Applied Microbiology, National Research and Innovation Agency, Bogor, Indonesia; ^3^Research Center for Pharmaceutical Ingredients and Traditional Medicine, National Research and Innovation Agency, Serpong, Indonesia; ^4^Research Center for Ecology and Ethnobiology, National Research and Innovation Agency, Bogor, Indonesia

**Keywords:** medicinal plant, benzoin resin, antifungal, antioxidant, *Trichoderma*, *Phyllosticta*, *Neopestalotiopsis*

## Abstract

Benzoin resin, produced by the native Indonesian trees *Styrax sumatrana* and *Styrax benzoin*, has been incorporated into medical practices to treat wounds, erythema, and many other conditions for centuries. Endophytic fungi that reside within medicinal plants have antimicrobial, antioxidant, and α-glucosidase inhibitory capacities, contributing to plant health and derivative products. In this study, we determined the antifungal, antioxidant, and α-glucosidase inhibitory capacities of endophytic fungal isolates from three different tissues (leaves, bark, and stems) of *S. sumatrana* and *S. benzoin* trees. The genera of fungal isolates were determined by phylogenetic analysis of internal transcribed spacer sequences. A total of 58 fungal isolates were classified into 15 different fungal genera from eight taxonomic orders—Hypocreales, Botryosphaeriales, Glomerellales, Diaphortales, Pleosporales, Eurotiales, Xylariales, and Mucorales—with a pattern of host species specificity. Among these isolates, *Trichoderma* sp. 6407 consistently exhibited high inhibition of the growth of plant pathogens *Fusarium* sp., *Trichoderma viride*, and *Aspergillus niger*. With respect to antioxidant activity, *Phyllosticta* sp. 6454 consistently showed 2,2-diphenyl-1-picrylhydrazyl inhibition (37.59 ± 0.05%), 2,2′-azino-bis (3-ethylbenzothiazoline-6-sulphonic acid)-based antioxidant activity (25.04 ± 0.27 mgTE/g), and α-glucosidase inhibitory activity (52.15 ± 10.08%). *Neopestalotiopsis* sp. 6431 was notably potent in 2,2-diphenyl-1-picrylhydrazyl inhibition (49.65 ± 0.80%), ferric reducing antioxidant power-based antioxidant activity (197.49 ± 8.65 mgTE/g), and α-glucosidase inhibitory activity (52.88 ± 4.93%). This study revealed that *Trichoderma* sp. 6407, *Phyllosticta* sp. 6454, and *Neopestalotiopsis* sp. 6431 exhibited antifungal, antioxidant, and α-glucosidase inhibitory activities.

## Introduction

Humankind has long used fragrances for health, beauty, and ceremonial purposes. The burning of fragrant incense has been observed in numerous cultures and religious ceremonies (Nir, 2004; Smit, 2004; Baum, 2013; Ergin, 2014; le Maguer, 2015; Milburn, 2016; Gershon, 2019). Ancient Greek, early Christian, Jewish, and Islamic societies used or use various fragrances, including balsamic resin, in their ritual practices (Haran, 1960; Nir, 2004; Smit, 2004; Ergin, 2014; le Maguer, 2015; Gershon, 2019). Balsamic resin has been traditionally used in Chinese culture for ceremonial and medicinal purposes, and the tradition has spread to other Asian countries (Milburn, 2016). Such practices have made the substance a valuable trading commodity (Kashio and Johnson, 2001; Ergin, 2014; le Maguer, 2015).

*Styrax* trees produce a balsamic resin known as benzoin resin (Kashio and Johnson, 2001). *Styrax sumatrana* and *Styrax benzoin* are native Indonesian trees that are widely cultivated in North Sumatra, Indonesia, where their resin has been traditionally used for herbal remedies and cultural ceremonies. For such applications, benzoin resin is valuable as a nontimber forest product (Nurwahyuni et al., 2021). Benzoin resin and its derivatives are incorporated into incense, cosmetics, and pharmaceutical products for their anti-inflammatory, antioxidant, and antimicrobial properties (Sharif et al., 2016; Hidayat et al., 2018, 2019b). The radical scavenging activity of resin from *S. sumatrana* has been reported to have high potency as an antioxidant; hence, it is a good candidate as a natural antioxidant resource (Nurwahyuni et al., 2021). Despite supporting the livelihood of 70% of local people in North Sumatra, Indonesia (Iswanto et al., 2016), and their value as a natural antioxidant resource, *Styrax* plantations for benzoin resin have gradually decreased because of land conversion (Saputra and Lee, 2021). Such limitations in plantation areas necessitate innovations in the sustainable production of benzoin resins and their derivatives.

In most plants, endophytic fungi that colonize the inner tissue play significant ecological roles, such as strengthening plant defenses against pathogens and abiotic stressors (Arnold et al., 2003; Schardl et al., 2004; Mejía et al., 2008). Particularly, in medicinal plants, these fungi have been reported to have distinctive relationships with their hosts; they influence the plant’s secondary metabolite production and antioxidant enzyme functioning and even incorporate their own metabolites into the host plant’s tissues, which consequently enhances the plant’s ability to withstand stress (Zhao et al., 2010; Ogbe et al., 2020). However, whether benzoin resin is synthesized and accumulated by endophytic fungi that reside within these plants remains an intriguing question.

Naturally sourced antioxidants have desirable properties that may reduce the use of chemically synthesized additives in food products (Brewer, 2011). The carcinogenic potential and other health risks of synthetic additives have limited their use and instigated the search for natural antioxidants (Capitani et al., 2013). A similar trend has been observed for cosmetics and pharmaceutical products. If the capacity of such functional substances from endophytic fungi is better than that of the host plant, endophytic fungi could be a more manageable and sustainable source option. Endophytic fungi isolated from medicinal plants, such as *Pinus roxburghii, Ginko biloba, Rauwolfia tetraphylla*, and agarwood-producing trees *Aquilaria* and *Gyrinops* have been reported to have antimicrobial and antioxidant capacities (Xiao et al., 2013; Bhardwaj et al., 2015; Alurappa and Chowdappa, 2018). However, information on endophytic fungi from *S. sumatrana* and *S. benzoin* remains scarce (Elfiati et al., 2021; Slamet et al., 2021). Considering the well-known role of endophytic fungi in enhancing plant defense against pathogens and the antioxidant function of benzoin resin (Hidayat et al., 2018), this study aimed to determine the antifungal, antioxidant, and alpha-glucosidase inhibitory activities of endophytic fungi isolated from the leaves, stems, and bark of *S. sumatrana* and *S. benzoin* trees.

## Materials and methods

### Sample collection and endophytic fungal isolation

Asymptomatic (healthy) 25- to 30-year-old *S. benzoin* and *S. sumatrana* trees with heights between 15 and 20 m and breast-high diameters between 15 and 25 cm were selected for this study (Slamet et al., 2021). Fragments were collected from plantations in North Sumatra Province, Indonesia, at elevations of 800–1,000 m asl and temperatures between 15 and 24°C. Tree fragments or plant organs (leaves, stems, or bark) were surface sterilized, and their inner parts were cut and planted aseptically on four chloramphenicol-supplemented isolation media: potato dextrose agar, Pachlewski, yeast dextrose agar, and yeast malt extract (Atlas, 2004; Hidayat et al., 2019a, 2021a); thereafter, successful endophytic fungal isolation was validated (Hidayat et al., 2019a,2021a). The obtained fungal isolates were deposited in the Indonesian Tropical Forest Culture Collection.

### Molecular identification, phylogenetic, and clustering analyses

Genomic DNA was extracted from a 7-day-old mycelial culture grown in potato dextrose broth with a DNA Wizard Kit (Promega, Madison, WI, USA) using the manufacturer’s method. Polymerase chain reaction (PCR) with Go Taq Green Master Mix (Promega) and ITS1 and ITS4 primers (White et al., 1990) was used to amplify the internal transcribed spacer (ITS) region of fungal DNA. PCR products were subjected to Sanger sequencing (1st BASE Sequencing Service, Singapore) and sequences were aligned using the Basic Local Alignment Search Tool from the National Center for Biotechnology Information database to identify closely related genera. The closest genera and number of isolates are referred to sequentially hereinafter as the isolate identities. A phylogenetic tree was constructed using Mega 11 (Tamura et al., 2021) and the neighbor-joining method (Saitou and Nei, 1987; Tamura et al., 2004). All sequences were deposited in the NCBI database with accession numbers ON796950 to ON97007 (Supplementary Table 1).

Single linkage with combined rescaled distance was calculated to observe the clustering of endophytic fungal genera based on the combination of host tree species and plant organs. Binary values were standardized before analysis. A dendrogram was constructed using hierarchical cluster analysis in IBM SPSS Statistics 25 (IBM Corp., Armonk, NY, USA) to determine relationships among endophytic fungi based on their origins. Squared Euclidean distance was used to express the cluster distance (Mufarhatun et al., 2021).

### Antifungal assay

A dual culture assay was used to screen fungal isolates for their ability to suppress mycelial growth of the plant pathogenic fungi *Fusarium* sp. (INTROF CC 0509) and *Trichoderma viride*, and *Aspergillus niger*. *T. viride*, and *A. niger* cultures were obtained from IPB University Culture Collection. Five-millimeter-diameter agar discs of endophytic and pathogenic fungal cultures were co-inoculated 3 cm apart on potato dextrose agar plates (90 mm diameter) and incubated at 25°C for 7 days. The percentage of inhibition (*%I*) was calculated using the following formula:


(1) %I=[(Jc-Jt)/Jc]×100%

where *Jc* is the pathogen’s radial outward growth (control) and *Jt* is its radial growth in the direction of the endophytic fungi (Hajieghrari et al., 2010). Each treatment was repeated five times.

### Antioxidant and antidiabetic assays

Prior to phytochemical assays for bioactive compounds, fungal extracts were prepared according to the method described by Hidayat et al. (2019a), with three replicates for each assay. Antioxidant activities were determined using three approaches: 2,2-diphenyl-1-picrylhydrazyl (DPPH), 2,2′-azino-bis (3-ethylbenzothiazoline-6-sulphonic acid) (ABTS), and ferric reducing antioxidant power (FRAP).

Determination of antioxidant activity *via* DPPH depletion followed the protocol described by Hidayat et al. (2021b). The effective concentration at which 50% of the radicals were scavenged (IC_50_ value) was obtained by interpolation from the linear regression analysis (Dewi et al., 2013). Concentrations of DPPH in ppm (based on IC_50_ estimation, *E*) were ranked as follows: *E* < 50, very strong; 50 ≤ *E <* 100, strong; 100 ≤ *E <* 150, moderate; 150 ≤ *E <* 200, weak; and ≥200, undetected.

Measurement of antioxidant activity using the ABTS approach was based on Trolox equivalent antioxidant capacity. The activity was expressed as Trolox equivalent in fungal extracts (mg TE/g), where the capacity of the sample to neutralize ABTS radicals is equivalent to that of Trolox (Wang et al., 2013). ABTS radical solution was prepared as follows: ABTS solution (7 mM) was mixed with potassium persulfate (140 mM) at a 62.5:1 ratio for the stock solution. The mixture was then kept in a dark room at 25°C for 16 h. For measurement, an ABTS radical solution with a 0.7 absorbance value at 734 nm was prepared. The assay was performed by mixing sample solution (5 μL, 1 mg/mL) with 200 μL of ABTS radical solution. The mixture was kept in a dark room for 6 min before absorbance reading at 734 nm. A Trolox solution with a range of concentrations was used to construct the standard curve.

Antioxidant capacity was determined using the FRAP approach according to the methods described by Dudonné et al. (2009) with slight modifications. FRAP reagent was prepared with 300 mM acetate buffer (pH 3.6), 10 mM 2,4,6-tri(2-pyridyl)-s-triazine, and 20 mM FeCl_3_ in a 10:1:1 ratio. Fungal extracts (40 μL) were then mixed with 1,200 μL FRAP reagent and incubated at 37°C for 30 min. FRAP values were based on the standard Trolox calibration curve and expressed in mg TE/g.

Measurement of total flavonoid content was performed according to the protocol described by Tambe and Bhambar (2014) with some modifications. A volume of 0.1 mL of the sample (1,000 μg/mL) was added to 2.6 mL distilled water and 0.15 mL 5% NaNO_2_, mixed, and incubated for 5 min. The mixture was combined with 0.15 mL 10% AlCl_3_ 10%, stirred, and incubated for 6 min before being combined with 2 mL NaOH 1 N, mixed, and incubated at 25°C for 30 min. Absorbance was measured at 510 nm. Quercetin at concentrations of 50, 100, 250, 500, and 1,000 μg/mL was used to construct the standard curve. Flavonoid content was expressed as quercetin equivalents (mg QE/g).

Total phenolic content was measured as previously described (Tambe and Bhambar, 2014) with some modifications. A volume of 0.1 mL of the sample (1,000 μg/mL) was combined with 1.4 mL distilled water and 0.25 mL Folin–Ciocalteau reagent. The mixture was incubated for 8 min, combined with 0.75 mL 20% Na_2_CO_3_, stirred, and incubated for 2 h at 27°C. Absorbance was measured at 750 nm wavelength. Three replicates of gallic acid at concentrations of 20, 40, 60, 80, and 100 μg/mL were used to construct the standard curve. Phenolic content was expressed as gallic acid equivalents (mg GAE/g).

Fungal extracts were evaluated for α-glucosidase inhibitory activity as described in our previous report (Dewi et al., 2013). Inhibition of α-glucosidase activity was determined by the reaction of α-glucosidase with the p-nitrophenyl-α-D-glucopyranoside substrate, resulting in the formation of p-nitrophenol (405 nm). Each assay was performed in triplicate, and the results are expressed as percentages of inhibition [% inhibition = (AB–AS)/AB × 100%], where AB is the absorbance of the blank solution and AS is the absorbance of the sample. Values are presented as the mean ± standard deviation.

### Liquid chromatography–high resolution mass spectrometer analysis

Secondary metabolites from the fungal extracts were determined using a Xevo G2-XS Quadrupole Time of Flight Mass Spectrometer (Waters Corp., Milford, MA, USA) equipped with an electrospray ionization source coupled to a Waters Acquity Ultra Performance LC system. Approximately, 1.5 mg of fungal extract was added to MeOH (LiChrosolv, hypergrade for LC–MS, Merck KGaA, Darmstadt, Germany) and sonicated for 10 min until completely dissolved. Samples were then filtered through a 0.22-μm polytetrafluoroethylene syringe filter (Waters Corp.) to obtain a final concentration of 1 mg/mL. LC–HRMS analysis was performed as described previously (Dewi et al., 2022). Bioactive compounds were identified and analyzed using the LC-HRMS and UNIFI software version 1.5. Peaks were tentatively assigned with the comparison to water built-in library.

### Statistical analysis

All results are presented as the mean of triplicate measurements and the standard deviation.

## Results

### Fungi isolated from *Styrax sumatrana* and *Styrax benzoin*, their molecular phylogeny, and origin-based clustering

Fifty-eight fungal isolates were obtained from the bark, stems, and leaves of *S. sumatrana* and *S. benzoin* trees. Thirty-eight isolates were obtained from 14 bark, eight stem, and nine leaf samples of *S. sumatrana* grown in four types of isolating media. Twenty isolates from four bark, nine stem, and seven leaf samples of *S. benzoin* were obtained using the same set of agar media.

**FIGURE 1:**
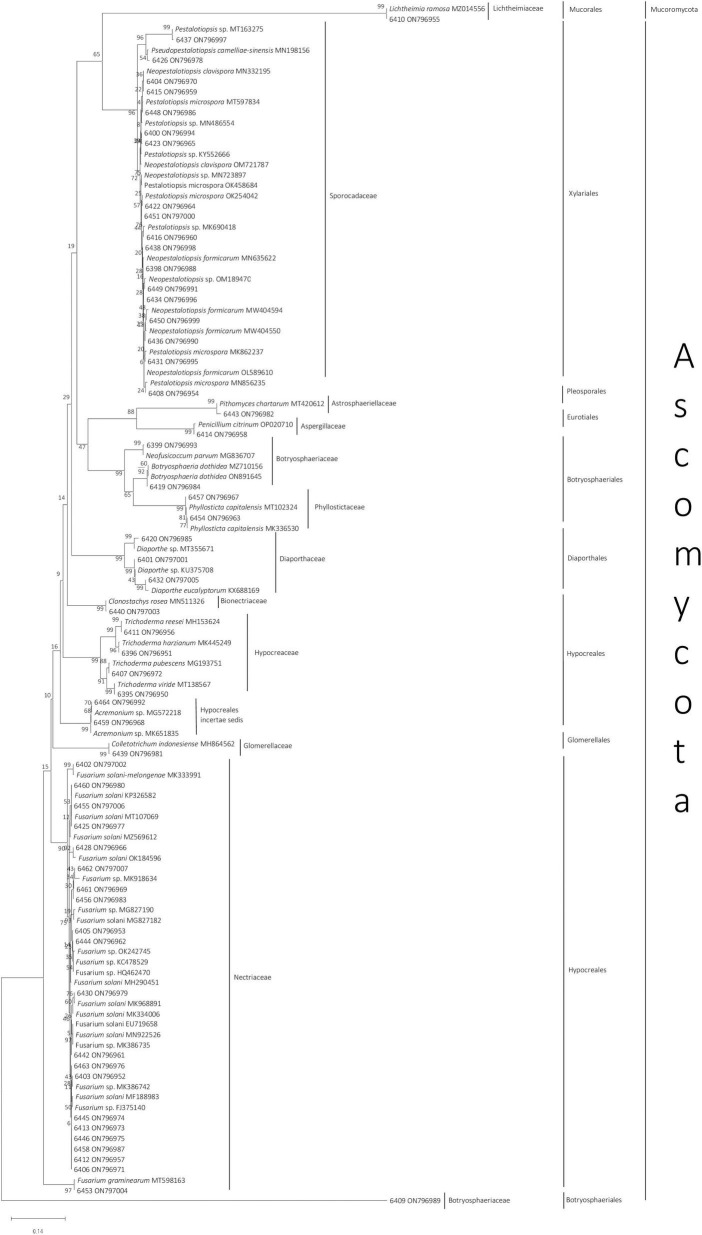
Neighbor-joining phylogenetic tree of 58 endophytic fungi isolated from *Styrax sumatrana* and *Styrax benzoin* and their respective reference based on fungal internal transcribed spacer sequence. Fungi from this study are presented as their isolate number followed by their National Center for Biotechnology Information accession number, whereas for reference sequences, species name, and accession number are given (Supplementary Table 1).

The genera of all 58 isolates were determined based on ITS sequences. The closest matching genera based on sequence similarity are shown in Figure 1. Almost all isolates belonged to the phylum Ascomycota, except *Lichtheimia* sp. 6410, which belonged to the phylum Mucoromycota. The 58 fungi identified in this study were classified into 15 genera belonging to 12 families that belong to eight orders: Hypocreales, Botryosphaeriales, Glomerellales, Diaphortales, Pleosporales, Eurotiales, Xylariales, and Mucorales (Figure 1). *Fusarium, Pestalotiopsis*, and *Neopestalotiopsis* were the most common genera, with 20, 9, and 8 isolates, respectively.

**FIGURE 2:**
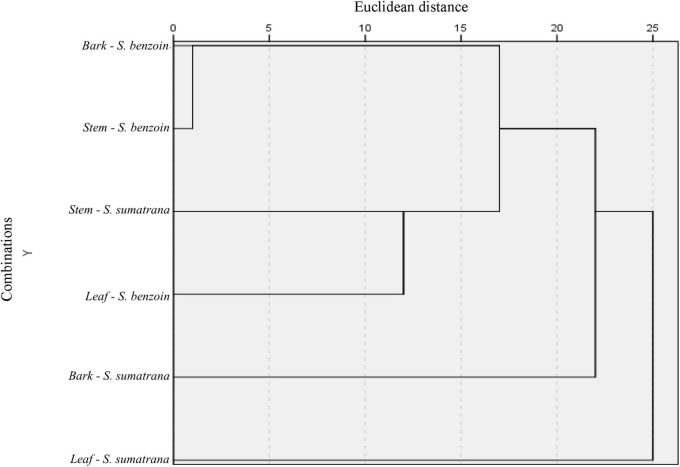
Single-linkage dendrogram with combined rescaled distance clusters. Endophytic fungi species were clustered based on their origins, plant organ (bark, stem, and leaf), and host plant species (*Styrax sumatrana* and *Styrax benzoin*).

Six possible combinations of two variables (host species and host organ) from which endophytic fungal genera originated are presented in Figure 2. A shorter Euclidean distance indicates a closer relationship; that is, the two variables share more similarities. Conversely, a longer Euclidean distance indicates greater heterogeneity and fewer similarities. Clustering analysis of endophytic fungal species based on the combination of plant organs and host plant species revealed two large groups. Host plant species seemed to be more important than plant organs in determining the grouping. Fungal communities residing in the leaves, bark, and stem of *S. sumatrana* tended to have more similarities and were thus grouped into one distinct cluster. The other cluster consisted of fungal communities residing in the leaves, bark, and stem of *S. benzoin*. A slightly different pattern was observed in each host plant species: in *S. benzoin*, fungal diversities in bark and stem organs showed more similarities to those residing in leaves, whereas in *S. sumatrana*, fungal diversities in bark and leaves showed more similarities to each other than to those in the stem.

### Antagonistic activity of endophytic fungal isolates against plant pathogenic fungi

All 58 endophytic fungal isolates exhibited *in vitro* growth inhibition of three plant-pathogenic fungi. The percentage inhibition ranged from 10.27 to 78.59%, 18.33 to 82.67%, and 6.31 to 73.57% for *Fusarium* sp., *T. viride*, and *A. niger*, respectively (Supplementary Table 2). Three most inhibiting isolates against *Fusarium* sp. were *Trichoderma* sp. 6395, *Trichoderma* sp. 6411, and *Trichoderma* sp. 6407, with averages of 78.59 ± 1.03%, 74.41 ± 3.61%, and 73.57 ± 7.56% inhibition, respectively. *Trichoderma* sp. 6407, *Fusarium* sp. 6413, and *Fusarium* sp. 6445 were the most inhibiting against *T. viride*, with 82.67 ± 4.94%, 76.00 ± 2.79%, and 69.00 ± 5.35% inhibition, respectively. *A. niger*, *Trichoderma* sp. 6407, *Neofusicoccum* sp. 6399, and *Trichoderma* sp. 6396 were the most inhibiting (73.57 ± 7.56%, 62.66 ± 3.25%, and 59.77 ± 10.50%, respectively) (Table 1).

**TABLE 1:** The high antagonistic activity of endophytic fungal isolates against plant pathogenic fungi, *Fusarium sp, Trichoderma viride, and Aspergillus niger* using dual culture assay*.

				Pathogenic fungal growth inhibition (%)**		
Host plant	Plant organ	Isolate number	Closest genus	*Fusarium sp.*	*Trichoderma viride*	*Aspergillus niger*
*Styrax sumatrana*	Bark	6395	*Trichoderma*	**78.59 ± 1.03**	38.67 ± 3.80	48.00 ± 13.83
		6396	*Trichoderma*	35.27 ± 19.14	58.67 ± 4.47	**59.77 ± 10.50**
		6411	*Trichoderma*	**74.41 ± 3.61**	63.33 ± 1.18	53.63 ± 14.39
	Stem	6407	*Trichoderma*	**73.57 ± 7.56**	**82.67 ± 4.94**	**73.57 ± 7.56**
		6413	*Fusarium*	65.00 ± 1.60	**76.00 ± 2.79**	55.10 ± 4.98
		6445	*Fusarium*	63.43 ± 2.10	**69.00 ± 5.35**	44.49 ± 14.38
*Styrax benzoin*	Stem	6399	*Neofusicoccum*	56.99 ± 18.96	59.00 ± 4.01	**62.66 ± 3.25**

*A complete list of 58 endophytic fungal isolates against plant pathogenic fungi is presented in Supplementary Table 2; **values are presented as mean ± standard deviation, which were performed in triplicate. The three isolates that showed the highest values of pathogenic fungal growth inhibition are presented in bold font.

**FIGURE 3:**
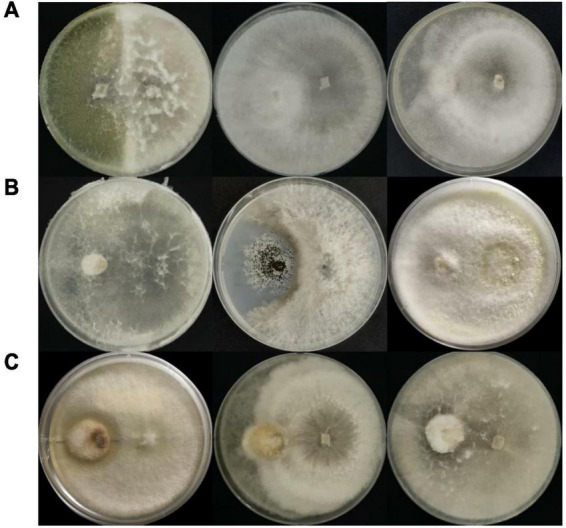
Inhibition of pathogenic fungi (placed on the left side of the plate); inhibition of **(A)** *Trichoderma viride*, **(B)** *Aspergillus niger*, and **(C)** *Fusarium* sp. by endophytic fungi (placed on the right side of the plate) in dual culture plates. The three highest inhibition percentages for each pathogen were obtained from isolates (left to right): **(A)** 6407, 6413, and 6445; **(B)** 6407, 6399, and 6396; **(C)** 6395, 6411, and 6407.

Corroborating Wheeler and Hocking (1993), *Trichoderma* sp. 6407, which inhibited all three pathogenic fungi, displayed interaction type B against *T. viride*, where both fungi inhibited each other’s growth and created less than 2 mm space between their colonies (Figure 3A, left). Both *Fusarium* spp. 6413 and 6445 exhibited interaction type C against *T. viride*, where the growth of each endophytic fungus was lower than that of the pathogen, followed by slower fungal growth when a barrier between colonies was apparent (Figure 3A, center and right). *Neofusicoccum* sp. 6399 and *A. niger* pathogen appeared to have type D interaction, where both cultures inhibited each other and created a space of 2 mm or more between colonies (Figure 3B, center). Interaction type E was observed in *Trichoderma* sp. 6395 and pathogenic *Fusarium* sp. dual cultures, where the pathogen’s growth was smaller and covered by endophytic fungi; however, the growth of both fungi decreased, and a barrier between colonies was apparent (Figure 3C, left). Interaction type F was observed in the remaining dual cultures, where pathogen growth was smaller than that in the endophytic fungal cultures, which later covered the pathogen’s colony (Figure 3C, center and right, and Figure 3B, left and right).

### Antioxidant activity of endophytic fungal extracts

Antioxidant activities of the endophytic fungal extracts were measured using DPPH, ABTS, and FRAP assays (Supplementary Table 2). Seven isolates with the highest values were exclusive for each method. The remaining three isolates were able to perform among the highest levels for the two methods. *Neopestalotiopsis* sp. 6431 extract performed the best in DPPH inhibition, with an average of 49.65 ± 0.80% or IC_50_ estimated concentration of 100.71 ppm, followed by *Trichoderma* sp. 6395, *Phyllosticta* sp. 6454, and *Fusarium* sp. 6430 extracts, tied at 37.59% or IC_50_ estimated concentration of 133.02 ppm. As previously mentioned, *Trichoderma* sp. 6395 was the most inhibiting in the dual culture assay against pathogenic *Fusarium* sp.

Estimated IC_50_ concentrations were all categorized as medium. For ABTS-based antioxidant activity, isolate 6412 extract led to an average of 28.41 ± 0.04 mg TE/g, closely followed by *Fusarium* sp. 6430 and *Phyllosticta* sp. 6454 extracts, with averages of 27.74 ± 0.34 and 25.04 ± 0.27 mg TE/g, respectively. The last two isolates were among the highest achievers of DPPH inhibition. *Neopestalotiopsis* sp. 6431, *Neopestalotiopsis* sp. 6450, and *Neofusicoccum* sp. 6399 were the three isolates with the highest antioxidant activities for the FRAP method: 197.49 ± 8.65, 167.39 ± 8.57, and 152.05 ± 32.40 mg TE/g, respectively. Moreover, the *Neopestalotiopsis* sp. 6431 extract exhibited the highest activity for both the DPPH and FRAP methods (Table 2).

**TABLE 2:** Fungal isolates with high antioxidant activity determined by 2,2-diphenyl-1-picrylhydrazyl (DPPH), 2,2′ -azino-bis (3-ethylbenzothiazoline-6-sulphonic acid) (ABTS), and ferric reducing antioxidant power (FRAP) assay*.

				Antioxidant activity**			
Host plant	Plant organ	Isolate number	Closest genus	DPPH			
				Inhibition (%)***	IC_50_ (ppm)	ABTS (mg TE/g)	FRAP (mgTE/g)
*Styrax sumatrana*	Bark	6395	*Trichoderma*	**37.59 ± 0.24**	**133,02**	2.72 ± 0.06	15.27 ± 0.16
		6412	*Fusarium*	26.24 ± 11.89	190,54	**28.41 ± 0.04**	93.99 ± 3.86
		6454	*Phyllosticta*	**37.59 ± 0.05**	**133,02**	**25.04 ± 0.27**	36.37 ± 2.22
	Stem	6430	*Fusarium*	**37.59 ± 0.56**	**133,02**	**27.74 ± 0.34**	40.20 ± 7.69
*Styrax benzoin*	Stem	6399	*Neofusicoccum*	ND	ND	22.21 ± 0.89	**152.05 ± 32.40**
		6431	*Neopestalotiopsis*	**49.65 ± 0.80**	**100,71**	3.35 ± 0.14	**197.49 ± 8.65**
		6450	*Neopestalotiopsis*	7.27 ± 6.98	687,41	18.71 ± 1.36	**167.39 ± 8.57**

*A complete list of 58 endophytic fungal isolates against plant pathogenic fungi is presented in Supplementary Table 2; **values are presented as mean ± standard deviation, which were performed in triplicate. Three isolates that achieved the highest values of antioxidant activity are presented in bold font are presented in bold font; ***the final concentration of each fungal extracted was applied at 100 μg/mL; ND: not detected; TE: Trolox equivalent.

An entirely different set of isolates, *Colletotrichum* sp. 6439, *Fusarium* sp. 6456, and *Fusarium* sp. 6444, produced extracts that contained the three highest concentrations of flavonoid compounds (155,79 ± 7.47, 108.86 ± 94.86, and 88.86 ± 14.49 mg QE/g, respectively) (Table 3). As for the total phenolic compounds, the three highest producers were fungal extracts from *Phyllosticta* sp. 6454, *Neopestalotiopsis* sp. 6431, and *Fusarium* sp. 6430 (110.87 ± 18.52, 43.02 ± 1.25, and 41.33 ± 1.50 mg GAE/g, respectively). These isolates were also the most inhibiting in the DPPH-based antioxidant capacity assay (Table 2).

**TABLE 3:** Fungal isolates with high content of flavonoid by using aluminum chloride assay, and phenol by using Folin–Ciocalteau reagent assay*.

				Phytochemical assay **	
Host plant	Plant organ	Isolate number	Closest genus	Flavonoid (mg QE/g)	Phenol (mg GAE/g)
*Styrax sumatrana*	Bark	6444	*Fusarium*	**88.86 ± 14.49**	27.10 ± 1.33
		6454	*Phyllosticta*	47.88 ± 8.52	**110.87 ± 18.52**
	Stem	6430	*Fusarium*	30.72 ± 7.00	**41.33 ± 1.50**
	Leaf	6439	*Colletotrichum*	**155.79 ± 7.47**	3.88 ± 0.20
		6456	*Fusarium*	**108.86 ± 94.86**	6.21 ± 0.32
*Styrax benzoin*	Stem	6431	*Neopestalotiopsis*	33.52 ± 0.28	**43.02 ± 1.25**

*A complete list of 58 endophytic fungal isolates against plant pathogenic fungi is presented in Supplementary Table 2; **values are presented as mean ± standard deviation, which were performed in triplicate; QE: quercetin equivalent; GAE: gallic acid equivalent. The three isolates that achieved the highest values in the phytochemical assay are presented in bold font.

### α-glucosidase inhibitory activity

α-glucosidase inhibitory activity of the 58 endophytic fungal isolates ranged from 0.00 to 65.00% (Supplementary Table 2). Fungal extracts of *Pestalotiopsis* sp. 6416, *Neopestalotiopsis* sp. 6431, and *Phyllosticta* sp. 6454 were the most inhibiting at 65.00 ± 0.28%, 52.88 ± 4.93%, and 52.15 ± 10.08%, respectively (Table 4). *Phyllosticta* sp. 6454 repeatedly resurfaced among the top three isolates for DPPH inhibition, ABTS-based antioxidant capacity, phenolic content, and α-glucosidase inhibitory activity. Similarly, *Neopestalotiopsis* sp. 6431 was the top performer in four assays: DPPH inhibition, FRAP-based antioxidant capacity, phenolic content, and α-glucosidase inhibitory activity (Tables 2–4).

**TABLE 4:** Fungal isolates with high α-glucosidase inhibition determined by p-nitrophenyl-α-D-glucopyranoside as substrate*.

Host plant	Plant organ	Isolate number	Closest genus	α-glucosidase inhibition (%)**
*Styrax sumatrana*	Bark	6416	*Pestalotiopsis*	65.00 ± 0.28
		6454	*Phyllosticta*	52.15 ± 10.08
*Styrax benzoin*	Stem	6431	*Neopestalotiopsis*	52.88 ± 4.93

*A complete list of 58 endophytic fungal isolates active against plant pathogenic fungi are presented in Supplementary Table 2; ** values are presented as mean ± standard deviation and were performed in triplicate for each fungal extract at a final concentration of 100 μg/mL.

### Detection of bioactive compounds

**FIGURE 4:**
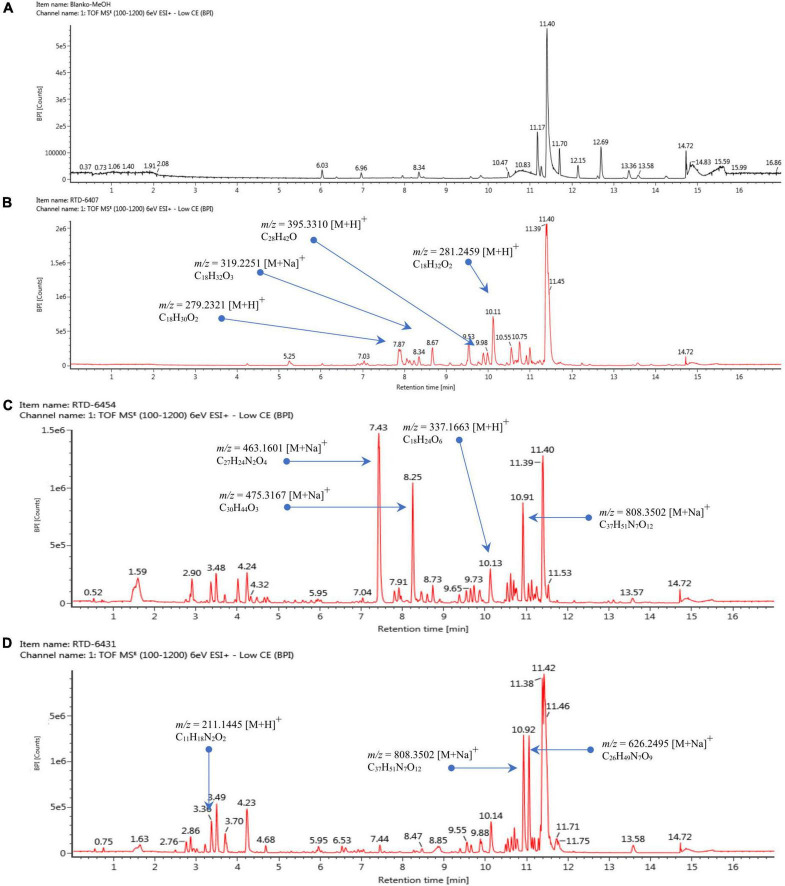
Liquid chromatography–mass spectrometry chromatogram of baseline **(A)**, extracted isolates *Trichoderma* sp. 6407 **(B)**, *Phyllosticta* sp. 6454 **(C)**, and *Neopestalotiopsis* sp. 6431 **(D)**.

**FIGURE 5:**
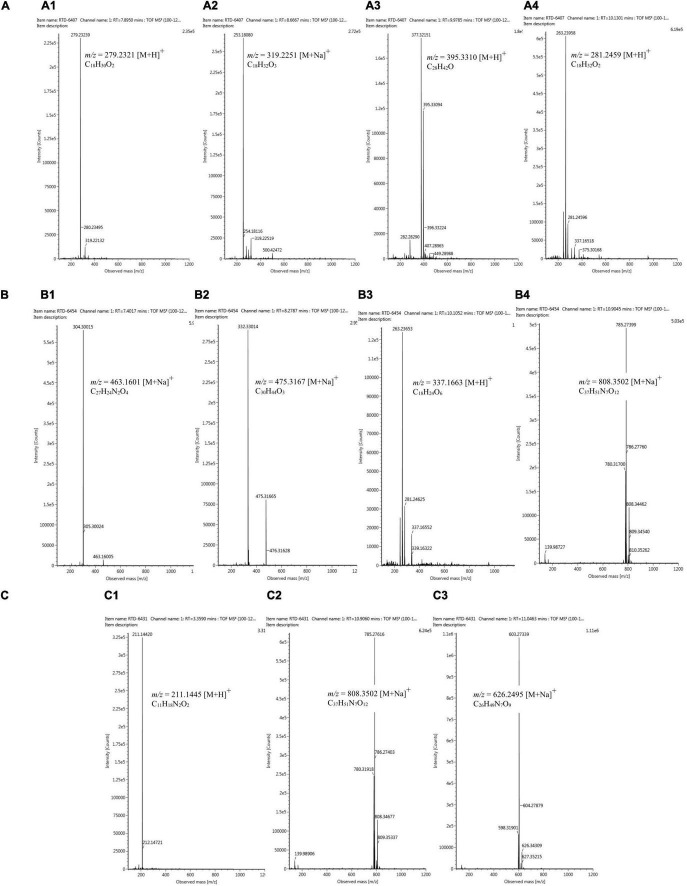
Mass chromatogram of metabolite compounds from extracted isolates *Trichoderma* sp. 6407 **(A1–4)**, *Phyllosticta* sp. 6454 **(B1–4)**, and *Neopestalotiopsis* sp. **(C1–3)**.

Based on the LC-HRMS results from base line (Figure 4A) and fungal crude extracts (Figures 4B–D), the crude extract of *Trichoderma* sp. 6407, which showed prominent antifungal activities, had eight peaks in total, with four main peaks indicating the active compounds (Figure 4B). The first four major peaks occurred at a retention time (*t*_R_) of 7.87 min, with a molecular ion (*m/z*) value of 279.2321 [M+H]^+^, and molecular formula C_18_H_30_O_2_. Bioactive compounds with such properties were predicted to be methyl hydroxysterpurate ethylidene acetal or xylarinorditerpene Q (Figures 4B, 5A1). The second four major peaks occurred at *t*_R_ = 8.67 min, with a *m/z* value of 319.2251 [M+H]^+^, and molecular formula C_18_H_32_O_3_, which could be (1S,2S)-3-oxo-2-pentylcyclopentane-1-octanoic acid or 3,7-dimethyl-9-(-2,2,5,5-tetramethyl-1,3-dioxolan-4-yl)nona-1,6-dien-3-ol (Figures 4B, 5A2). The third four major peaks occurred at *t*_R_ = 9.98 min, with an *m/z* value of 395.3310 [M+H]^+^, and molecular formula C_28_H_42_O, which were predicted to be ergosta-4,6,8,22E-tetraen-11β-OL (Figures 4B, 5A3). The last detected major peak occurred at t_R_ = 10.91 min, with a predicted *m/z* value of 281.2459 [M+H]^+^, and molecular formula C_18_H_32_O_2_, which were approximated to be 4-Me-6E,8E-16:2 methyl ester or 4-methyl-7,11-heptadecadienoic acid (Figures 4B, 5A4).

Several ionic predictions and molecular formulas for *Phyllosticta* sp. 6454 extracts, which showed antioxidant and α-glucosidase activity, were identified (Figures 4C, 5B). The LC–HRMS results showed at least 10 peaks for active compounds. The four largest peaks had a *t*_R_ of 7.43, 8.25, 10.13, and 10.19 min (Figure 4C), with the following respective *m/z* values and molecular formulas: *m/z* = 463.1601 [M+Na]^+^ and molecular formula C_27_H_24_N_2_O_4_ (predicted to be SF2809-IV (4-hydroxy-3-[[3-(2-hydroxyethyl)-1H-indol-2-yl]-(4-hydroxyphenyl)methyl]-1-methylquinolin-2-one) compound); *m/z* = 475.3167 [M+Na]^+^ and molecular formula C_30_H_44_O_3_ (predicted to be igniarine); *m/z* = 337.1663 [M+H]^+^ and molecular formula C_18_H_24_O_6_ (predicted to be corynechromone or cytosprone); and *m/z* = 808.3502 [M+Na]^+^ and molecular formula C_37_H_51_N_7_O_12_ (Figures 5B1–4).

For the active compounds in *Neopestalotiopsis* sp. 6431 extracts, which may be responsible for the exhibited antioxidant activities, at least nine peaks were predicted (Figure 4D). The three largest peaks were detected at a t_R_ of 3.36, 10.92, and 11.05 min (Figure 4D), with the following respective *m/z* values and molecular formulas: *m/z* = 211.1445 [M+H]^+^ and molecular formula C_11_H_18_N_2_O_2_; *m/z* = 808.3502 [M+Na]^+^ and molecular formula C_37_H_51_N_7_O_12_; and *m/z* = 626.3495 [M+Na]^+^ and molecular formula C_26_H_49_N_7_O_9_ (Figures 5C1–3).

## Discussion

All the closest matches of the isolates belong to the phylum Ascomycota, except for *Lichtheimia*, which belongs to Mucoromycota. The dominance of Ascomycota in endophytic fungal assemblages has also been observed elsewhere (Hamzah et al., 2018; Zhou et al., 2018; Nguyen et al., 2021). This study complements previous reports on endophytic fungi isolated from *S. sumatrana* and *S. benzoin* and adds the newly reported genera *Lichtheimia, Penicillium, Pseudopestalotiopsis*, and *Colletotrichum* from *S. sumatrana* and *Botryosphaeria* from *S. benzoin* (Hidayat et al., 2021a; Slamet et al., 2021). All of the closest-matched genera have been previously reported as endophytes (Shetty et al., 2016; Huang et al., 2020). Various factors are involved in the dynamics of endophytic fungal communities, including macroenvironmental factors such as season, geographic location (Mishra et al., 2012; Slamet et al., 2021), and water availability (Costa et al., 2018). Each microenvironmental biotic factor, including host species, host tissue/organ (Moricca et al., 2012; Slamet et al., 2021), and other coexisting endophytes or pathogens (Sicard et al., 2007), adds to the community dynamics. These complex mechanisms may act as selection pressures for endophytic fungi, leading to host species and/or host organ specificities (Slamet et al., 2021).

Clustering the endophytic fungal genera based on the plant host species and plant organs demonstrated a specific pattern only for the plant host species. Endophytic fungal communities in the bark and stems of *S. benzoin* clustered within the shortest Euclidean distance, indicating higher genus similarity and homogeneity between them. The fungal community in the leaves of the same host species was clustered at a longer distance but was still within the same cluster. Although these organs have different structures (Romero, 2014), the closer proximity of the bark and stem may accommodate fungal mycelia extending between these two organs and consequently having more shared species. In the other cluster, the fungal community residing in the bark and leaf organs of *S. sumatrana* shared more similarities than those residing in the stems of the same host plant species. This pattern of similarities indicates the tendency of fungal genus specificity to be based on host plant species. These observations are based on the genus taxonomic rank, which is limited to a less restrictive interpretation. Multi-locus taxonomy identification and/or an inclusive metagenomic approach is recommended for further studies to scrutinize these mechanisms.

*Trichoderma* sp. 6407, isolated from the stems of *S. sumatrana*, consistently showed the highest antifungal activity against all tested pathogenic fungi. Plant-protective members of the *Trichoderma* genus, including *T. pubescens*, produce a group of polypeptide antibiotics that may contribute to their antagonistic potential against fungal diseases in grapevine trunks (Degenkolb et al., 2006). Other species of *Trichoderma* have been shown to reduce disease severity by inhibiting the growth of the pathogenic fungus *Rhizoctonia solani* (Molan et al., 2008). The predicted methyl hydroxysterpurate ethylidene acetal or xylarinorditerpene Q compounds from *Trichoderma* sp. 6407 extracts were previously reported to be isolated from endophytic fungi and also have antifungal properties (Xie and Li, 1992; Wu et al., 2014; Chen et al., 2020). The predicted (1S,2S)-3-oxo-2-pentylcyclopentane-1-octanoic acid or 3,7-dimethyl-9-(-2,2,5,5-tetramethyl-1,3-dioxolan-4-yl)nona-1,6-dien-3-ol compounds have also been reported to be produced by endophytic fungi (Miersch et al., 1999; Lin et al., 2016). The prediction ergosta-4,6,8,22E-tetraen-11β-OL compound has previously been reported to be isolated from the fruiting body of *Coprinus setulosus* (Ma et al., 2018), whereas, the postulated 4-Me-6E,8E-16:2 methyl ester or 4-methyl-7,11-heptadecadienoic acid compounds were reported to be isolated from liquid cultures of *Clonostachys rosea* and *Sporothrix* sp. and have the potential to inhibit the growth of MFC-7 cancer cells, *F. oxysporum* f. sp. *lycospersici, T. viride*, and *Bacillus subtilis* (Choudhury et al., 1994; Dias et al., 2015). In this study, the metabolites produced by *Trichoderma* sp. 6407 displayed four major peaks; however, other minor peaks still have the potential to represent other active compounds. Considering the vital role of endophytic fungi in strengthening plant defense against pathogens and promoting overall health (Mejía et al., 2008), purification and elucidation of the active compounds produced by *Trichoderma* sp. 6407 and further investigation of antifungal mechanisms are required to optimize their biocontrol potential.

*Phyllosticta* sp. 6454, which was isolated from the bark of *S. sumatrana*, is characterized by its high antioxidant and α-glucosidase activity. This genus is a widely distributed fungal endophyte and is found in 70 plant families (Wikee et al., 2013). Despite its widespread distribution, information regarding its antioxidant capacity is limited. Endophytic *Phyllosticta* sp. isolated from the medicinal plant *Guazuma tomentosa* has been reported to have antioxidant properties, with phenol and flavonoid contents that are one-sixth and one-fourteenth, respectively, of those observed in *Phyllosticta* sp. 6454 (Srinivasan et al., 2010). The presumed SF2809-IV (4-hydroxy-3-[[3-(2-hydroxyethyl)-1H-indol-2-yl]-(4-hydroxyphenyl)methyl]-1-methylquinolin-2-one) compound from *Phyllosticta* sp. 6454 extracts were previously isolated from *Dactylosporangium* sp., and some of its derivatives have been shown to inhibit recombinant human chymase activity (Tani et al., 2004). Secondary metabolites from *Phyllosticta* with antimicrobial activities have been previously reported (Taher et al., 2022) and may be related to the phenol and flavonoid contents of *Phyllosticta* sp. 6454 extracts.

*Neopestalotiopsis* sp. 6431, isolated from the stems of *S. benzoin*, also exhibited high antioxidant activity. *Neopestalotiopsis* is a common endophytic fungal genus (Hamzah et al., 2018; Azuddin et al., 2021) and was among the most commonly isolated genera in this study. This genus was also previously discovered in the bark of *S. benzoin* (Ilyas et al., 2019). A member of the genus *Neopestalotiopsis* has been reported to produce antimicrobial and antioxidant agents, such as eugenol, myristaldehyde, lauric acid, and caprylic acid (Tanapichatsakul et al., 2019). However, based on the LC–HRMS results of the present study, *Neopestalotiopsis* sp. 6431 may have produced different secondary metabolites.

*Fusarium* sp. 6430 isolated from the stems of *S. sumatrana* was the most frequently isolated genus in this study and displayed high antioxidant activity. This genus has been reported to exude diverse bioactive compounds and exert biocontrol functions to enhance plant health. Secondary metabolites, aza-anthraquinones, isolated from an endophytic *F. solani* strain, the crude extract of which shows antimicrobial and antioxidant activities, have been reported to be potent bioactive compounds for anticancer and antimicrobial agents (Khan et al., 2018). *Fusarium* sp. evinced the production of a new antifungal and antimalarial cyclodepsipeptide, known as fusaripeptide (Ibrahim et al., 2018). The observed antioxidant activity and phenol content of *Phyllosticta* sp. 6454, *Neopestalotiopsis* sp. 6431, and *Fusarium* sp. 6430 suggests that these isolates are strong candidates for natural antioxidant sources. Future studies, including methods of purifying bioactive compounds, are required to further optimize their potential as bioresources.

Antioxidants are compounds that inhibit the initiation or propagation of chain oxidation reactions. The chemical structure of antioxidants, source of free radicals, and physicochemical properties of different sample preparations can provide different test results for antioxidant activity (Karamać et al., 2005). Therefore, it is necessary to analyze the antioxidant activity of a specific sample type. In this study, antioxidant testing using DPPH, ABTS, and FRAP assays was conducted. The DPPH assay measures the ability of compounds to donate hydrogen to a stable DPPH• molecule, resulting in the formation of a purple color (520 nm). In contrast, the ABTS cation radical (ABTS•^+^), which absorbs light at 743 nm, changes to stable ABTS by accepting hydrogen from antioxidant compounds, resulting in solution decolorization (Chu et al., 2000). This method is based on a reduction reaction in an acidic atmosphere to a yellow Fe^3+^ (potassium hexacyanoferrate) complex compound to a bluish-green Fe^2+^ complex compound owing to electrons donated by antioxidant compounds (Craft et al., 2012).

Furthermore, we investigated the α-glucosidase inhibitory activity of the fungal extracts in this study. Phenolic compounds are antioxidants and can also inhibit natural α-glucosidase enzymes because they inhibit carbide enzymes owing to their ability to bind proteins (Zhang et al., 2015). This assay is based on the formation of p-nitrophenol, which results from the cleavage of p-nitrophenyl-α-D-glucopyranose at 410 nm.

These findings lay the groundwork for further studies that will identify the compounds responsible for the observed antioxidant and α-glucosidase inhibitory activities and their underlying mechanisms, which will potentially guide the optimization of their production.

## Data availability statement

The datasets presented in this study can be found in online repositories. The names of the repository/repositories and accession number(s) can be found in the article/Supplementary material.

## Author contributions

DE, SF, LR, AA, RD, HR, MT, MR, AS, and AH contributed to the conceptualization, methodology, experiment, validation, analysis, resources, writing, review, and editing of the manuscript. All authors have read and agreed to the published version of the manuscript.
