# Supplementary material for: Culturable endophytic fungal assemblages from Styrax sumatrana and Stryax benzoin and their potential as antifungal, antioxidant, and alpha-glucosidase inhibitory resources
Source: Front Microbiol. 2022 Nov 4;13:974526. doi: 10.3389/fmicb.2022.974526 (PMC9672939; doi:10.3389/fmicb.2022.974526)
Supplement: Supplementary file 1 [file Table_1.DOCX]

***Supplementary Material***

**Supplementary Table 1**. Endophytic fungi from *Styrax* sp. with their reference (closest species) based on fungal internal transcribed spacer (ITS) sequence that were aligned using basic local alignment search tool (BLAST)

| No | Fungi isolates | | | References | | | | |
| --- | --- | --- | --- | --- | --- | --- | --- | --- |
|  | Isolate number | NCBI accession number | Scientific name | | E Value | Match identity | NCBI accession number |  |
| 1 | 6395 | ON796950 | *Trichoderma viride* | | 0.0 | 99.8 | MT138567 |  |
| 2 | 6396 | ON796951 | *Trichoderma harzianum* | | 0.0 | 99.7 | MK445249 |  |
| 3 | 6403 | ON796952 | *Fusarium solani* | | 0.0 | 100.0 | MF188983 |  |
| 4 | 6405 | ON796953 | *Fusarium* sp. | | 0.0 | 99.8 | KC478529 |  |
| 5 | 6408 | ON796954 | *Neopestalotiopsis* sp. | | 0.0 | 100.0 | OM189470 |  |
| 6 | 6410 | ON796955 | *Lichtheimia ramosa* | | 0.0 | 100.0 | MZ014556 |  |
| 7 | 6411 | ON796956 | *Trichoderma reesei* | | 0.0 | 99.4 | MH153624 |  |
| 8 | 6412 | ON796957 | *Fusarium* sp. | | 0.0 | 99.8 | MK386742 |  |
| 9 | 6414 | ON796958 | *Penicillium citrinum* | | 0.0 | 100.0 | OP020710 |  |
| 10 | 6415 | ON796959 | *Neopestalotiopsis clavispora* | | 0.0 | 100.0 | OM721787 |  |
| 11 | 6416 | ON796960 | *Pestalotiopsis* sp. | | 0.0 | 100.0 | MK690418 |  |
| 12 | 6442 | ON796961 | *Fusarium solani* | | 0.0 | 99.8 | MN922526 |  |
| 13 | 6444 | ON796962 | *Fusarium* sp. | | 0.0 | 99.8 | HQ462470 |  |
| 14 | 6454 | ON796963 | *Phyllosticta capitalensis* | | 0.0 | 100.0 | MK336530 |  |
| 15 | 6422 | ON796964 | *Pestalotiopsis microspora* | | 0.0 | 100.0 | OK458684 |  |
| 16 | 6423 | ON796965 | *Pestalotiopsis* sp. | | 0.0 | 99.8 | KY552666 |  |
| 17 | 6428 | ON796966 | *Fusarium solani* | | 0.0 | 100.0 | OK184596 |  |
| 18 | 6457 | ON796967 | *Phyllosticta capitalensis* | | 0.0 | 100.0 | MT102324 |  |
| 19 | 6459 | ON796968 | *Acremonium* sp. | | 0.0 | 100.0 | MK651835 |  |
| 20 | 6461 | ON796969 | *Fusarium* sp. | | 0.0 | 99.8 | OK242745 |  |
| 21 | 6404 | ON796970 | *Neopestalotiopsis clavispora* | | 0.0 | 100.0 | MN332195 |  |
| 22 | 6406 | ON796971 | *Fusarium* sp. | | 0.0 | 99.5 | MK386735 |  |
| 23 | 6407 | ON796972 | *Trichoderma pubescens* | | 0.0 | 100.0 | MG193751 |  |
| 24 | 6413 | ON796973 | *Fusarium* sp. | | 0.0 | 99.6 | MK918634 |  |
| 25 | 6445 | ON796974 | *Fusarium solani* | | 0.0 | 99.6 | MK334006 |  |
| 26 | 6446 | ON796975 | *Fusarium* sp. | | 0.0 | 99.8 | FJ375140 |  |
| 27 | 6463 | ON796976 | *Fusarium solani* | | 0.0 | 99.6 | MH290451 |  |
| 28 | 6425 | ON796977 | *Fusarium solani* | | 0.0 | 100.0 | MT107069 |  |
| 29 | 6426 | ON796978 | *Pseudopestalotiopsis camelliae-sinensis* | | 0.0 | 99.3 | MN198156 |  |
| 30 | 6430 | ON796979 | *Fusarium solani* | | 0.0 | 100.0 | MK968891 |  |
| 31 | 6460 | ON796980 | *Fusarium solani* | | 0.0 | 100.0 | KP326582 |  |
| 32 | 6439 | ON796981 | *Colletotrichum indonesiense* | | 0.0 | 100.0 | MH864562 |  |
| 33 | 6443 | ON796982 | *Pithomyces chartarum* | | 0.0 | 100.0 | MT420612 |  |
| 34 | 6456 | ON796983 | *Fusarium* sp. | | 0.0 | 99.8 | MG827190 |  |
| 35 | 6419 | ON796984 | *Botryosphaeria dothidea* | | 0.0 | 99.8 | MZ710156 |  |
| 36 | 6420 | ON796985 | *Diaporthe* sp. | | 0.0 | 98.5 | MT355671 |  |
| 37 | 6448 | ON796986 | *Pestalotiopsis microspora* | | 0.0 | 100.0 | MT597834 |  |
| 38 | 6458 | ON796987 | *Fusarium solani* | | 0.0 | 99.6 | EU719658 |  |
| 39 | 6398 | ON796988 | *Pestalotiopsis microspora* | | 0.0 | 100.0 | OK254042 |  |
| 40 | 6409 | ON796989 | *Botryosphaeria dothidea* | | 0.0 | 99.8 | ON891645 |  |
| 41 | 6436 | ON796990 | *Neopestalotiopsis formicarum* | | 0.0 | 100.0 | MW404594 |  |
| 42 | 6449 | ON796991 | *Pestalotiopsis microspora* | | 0.0 | 100.0 | MK862237 |  |
| 43 | 6464 | ON796992 | *Acremonium* sp. | | 0.0 | 99.4 | MG572218 |  |
| 44 | 6399 | ON796993 | *Neofusicoccum parvum* | | 0.0 | 100.0 | MG836707 |  |
| 45 | 6400 | ON796994 | *Pestalotiopsis* sp. | | 0.0 | 100.0 | MN486554 |  |
| 46 | 6431 | ON796995 | *Neopestalotiopsis formicarum* | | 0.0 | 100.0 | MW404550 |  |
| 47 | 6434 | ON796996 | *Neopestalotiopsis formicarum* | | 0.0 | 100.0 | MN635622 |  |
| 48 | 6437 | ON796997 | *Pestalotiopsis* sp. | | 0.0 | 100.0 | MT163275 |  |
| 49 | 6438 | ON796998 | *Pestalotiopsis microspora* | | 0.0 | 100.0 | MN856235 |  |
| 50 | 6450 | ON796999 | *Neopestalotiopsis formicarum* | | 0.0 | 100.0 | OL589610 |  |
| 51 | 6451 | ON797000 | *Neopestalotiopsis* sp. | | 0.0 | 100.0 | MN723897 |  |
| 52 | 6401 | ON797001 | *Diaporthe* sp. | | 0.0 | 99.3 | KU375708 |  |
| 53 | 6402 | ON797002 | *Fusarium solani-melongenae* | | 0.0 | 100.0 | MK333991 |  |
| 54 | 6440 | ON797003 | *Clonostachys rosea* | | 0.0 | 100.0 | MN511326 |  |
| 55 | 6453 | ON797004 | *Fusarium graminearum* | | 0.0 | 100.0 | MT598163 |  |
| 56 | 6432 | ON797005 | *Diaporthe eucalyptorum* | | 0.0 | 99.8 | KX688169 |  |
| 57 | 6455 | ON797006 | *Fusarium solani* | | 0.0 | 99.6 | MZ569612 |  |
| 58 | 6462 | ON797007 | *Fusarium solani* | | 0.0 | 99.8 | MG827182 |  |
