# Supplementary material for: Culturable endophytic fungal assemblages from Styrax sumatrana and Stryax benzoin and their potential as antifungal, antioxidant, and alpha-glucosidase inhibitory resources
Source: Front Microbiol. 2022 Nov 4;13:974526. doi: 10.3389/fmicb.2022.974526 (PMC9672939; doi:10.3389/fmicb.2022.974526)
Supplement: Supplementary file 2 [file Table_2.docx]

Supplementary Material

| **Supplementary Table 2**. Antifungal and phytochemical assays of endophytic fungi isolated from *Styrax sumatrana* and *Styrax benzoin* | | | | | | | | | | | | | | | |
| --- | --- | --- | --- | --- | --- | --- | --- | --- | --- | --- | --- | --- | --- | --- | --- |
| **Host plant** | **Plant organ** | **Isolate number** | **Closest genus** | **Pathogenic fungal growth inhibition**  **(%)** | | | **Phytochemical assays** | | | | | | | | |
|  |  |  |  |  |  |  | **DPPH** | | **ABTS**  **mg TE/g)** | **FRAP**  **(mgTE/g)** | **Flavonoid**  **(mg QE/g)** | **Phenol**  **(mg GAE/g)** | **α-Glucosidase**  **inhibition**  **(%)** | | |
|  |  |  |  | ***Fusarium sp.*** | ***Trichoderma viride*** | ***Aspergillus niger*** | **Inhibition (%)** | **IC_50_**  **(ppm)** |  |  |  |  |  |  |  |
| ***Styrax sumatrana*** | **Bark** | 6395 | *Trichoderma* | **78.59 ± 1.03** | 38.67 ± 3.80 | 48.00 ± 13.83 | 37.59 ± 0.24 | **133,02** | 2.72 ± 0.06 | 15.27 ± 0.16 | 39.71 ± 10.25 | 16.86 ± 0.50 | 4.14 ± 0.02 | | |
|  |  | 6396 | *Trichoderma* | 35.27 ± 19.14 | 58.67 ± 4.47 | **59.77 ± 10.50** | 18.88 ± 12.07 | 264,79 | 10.09 ± 2.98 | 19.94 ± 0.11 | 47.02 ± 28.76 | 7.50 ± 0.60 | 14.65 ± 0.98 | | |
|  |  | 6411 | *Trichoderma* | **74.41 ± 3.61** | 63.33 ± 1.18 | 53.63 ± 14.39 | 3.94 ± 14.20 | 1269,23 | 14.84 ± 0.27 | 12.46 ± 12.79 | 57.45 ± 31.49 | 24.49 ± 1.20 | 3.87 ± 0.16 | | |
|  |  | 6412 | *Fusarium* | 60.71 ± 0.00 | 64.00 ± 3.46 | 32.11 ± 3.81 | 26.24 ± 11.89 | 190,54 | **28.41 ± 0.04** | 93.99 ± 3.86 | 13.12 ± 6.38 | 16.37 ± 0.98 | 12.89 ± 1.20 | | |
|  |  | 6416 | *Pestalotiopsis* | 67.62 ± 3.61 | 49.33 ± 2.79 | 43.56 ± 1.50 | 17.22 ± 12.24 | 290,44 | 3.54 ± 4.60 | 22.35 ± 0.69 | 9.04 ± 10.48 | 3.93 ± 0.20 | **65.00 ± 0.28** | | |
|  |  | 6444 | *Fusarium* | 63.33 ± 2.36 | 27.33 ± 6.41 | 39.85 ± 4.03 | 30.06 ± 0.38 | 166,35 | 9.95 ± 0.05 | 0.00 ± 0.00 | **88.86 ± 14.49** | 27.10 ± 1.33 | 2.71 ± 1.23 | | |
|  |  | 6403 | *Fusarium* | 60,43 ± 2.29 | 42.00 ± 1.83 | 33.79 ± 10.89 | ND | ND | 22.11 ± 0.74 | 16.05 ± 5.71 | 17.23 ± 6.05 | 40,96 ± 4.96 | 7.77 ± 0.33 | | |
|  |  | 6405 | *Fusarium* | 53.97 ± 5.77 | 36.00 ± 3.65 | 43.57 ± 5.04 | 19.83 ± 16,71 | 252,15 | 5.41 ± 1.80 | 9.56 ± 3.91 | 16.34 ± 5.37 | 4.29 ± 0.67 | 2.44 ± 1.13 | | |
|  |  | 6408 | *Neopestalotiopsis* | 66.19 ± 2.83 | 50.00 ± 0.00 | 47.92 ± 4.47 | 17.53 ± 12,16 | 285,23 | 21.13 ± 0.24 | 47.36 ± 0.19 | 21.33 ± 0.05 | 9.01 ± 1.09 | 7.18 ± 2.35 | | |
|  |  | 6414 | *Penicillium* | 51.30 ± 4.01 | 26.67 ± 7.82 | 47.57 ± 2.63 | 3.57 ± 14,33 | 1399,46 | 6.45 ± 1.59 | 15.92 ± 2.90 | 22.79 ± 0.30 | 4.65 ± 0.23 | ND | | |
|  |  | 6415 | *Neopestalotiopsis* | 68.65 ± 3.34 | 48.33 ± 2.36 | 43.08 ± 6.50 | 18.35 ± 7,35 | 272,55 | 8.79 ± 1.44 | 41.31 ± 1.57 | 55.55 ± 13.56 | 26.01 ± 1.06 | 5.03 ± 0.68 | | |
|  |  | 6442 | *Fusarium* | 60.67 ± 1.90 | 36.67 ± 4.71 | 42.29 ± 9.33 | 19.24 ± 5,23 | 259,83 | 24.68 ± 1.79 | 10.54 ± 0.68 | 29.76 ± 7.69 | 40.13 ± 1.34 | 3.93 ± 0.27 | | |
|  |  | 6422 | *Pestalotiopsis* | 70.98 ± 2.27 | 50.33 ± 4.77 | 54.86 ± 8.57 | ND | ND | 27.81 ± 0.01 | 24.62 ± 8.18 | 34.03 ± 5.77 | 20.88 ± 0.28 | 14.94 ± 2.96 | | |
|  |  | 6423 | *Pestalotiopsis* | 60.22 ± 1.68 | 41.00 ± 1.49 | 35.85 ± 4.31 | 3.64 ± 13,72 | 1373,46 | 3.78 ± 0.91 | 35.45 ± 7.36 | 15.87 ± 4.32 | 4.62 ± 0.52 | 2.45 ± 0.24 | | |
|  |  | 6428 | *Fusarium* | 52.78 ± 6.86 | 33.33 ± 0.00 | 40.41 ± 10.26 | 7.97 ± 20,22 | 627,42 | 17.82 ± 1.34 | 48.55 ± 8.30 | 49.30 ± 12.24 | 40.70 ± 4.09 | 19.35 ± 1.53 | | |
|  |  | 6457 | *Phyllosticta* | 18.01 ± 10.28 | 41.33 ± 3.98 | 33.41 ± 3.37 | 18.35 ± 7,80 | 272,55 | 2.67 ± 1.04 | 8.63 ± 3.38 | 10.20 ± 2.45 | 14.32 ± 1.23 | 5.55 ± 0.45 | | |
|  |  | 6459 | *Acremonium*. | 10.27 ± 5.07 | 24.67 ± 1.83 | 36.28 ± 2.23 | 11.87 ± 19,63 | 421,20 | 21.35 ± 2.13 | 62.18 ± 6.75 | 32.98 ± 5.29 | 36.57 ± 3.74 | 41.34 ± 1.08 | | |
|  |  | 6461 | *Fusarium* | 44.66 ± 11.59 | 37.67 ± 2.53 | 21.58 ± 11.14 | 29.57 ± 8,37 | 169,09 | 4.72 ± 1.53 | ND | 26.26 ± 6.26 | 15.31 ± 0.53 | 3.83 ± 0.46 | | |
|  |  | 6410 | *Lichtheimia* | 57.24 ± 2.01 | 35.67 ± 4.94 | 49.63 ± 5.30 | ND | ND | 3.51 ± 1.77 | 7.95 ± 0.17 | 16.39 ± 4.46 | 11.97 ± 1.97 | 1.23 ± 0.29 | | |
|  |  | 6454 | *Phyllosticta* | 56.84 ± 1.71 | 33.33 ± 0.00 | 36.20 ± 9.35 | 37.59 ± 0.05 | **133.02** | **25.04 ± 0.27** | 36.37 ± 2.22 | 47.88 ± 8.52 | **110.87 ± 18.52** | **52.15 ± 10.08** | | |
|  | **Stem** | 6407 | *Trichoderma* | **73.57 ± 7.56** | **82.67 ± 4.94** | **73.57 ± 7.56** | 23.48 ± 13.06 | 212,95 | 18.11 ± 1.05 | 31.53 ± 2.20 | 41.70 ± 14.57 | 25.95 ± 2.50 | 6.85 ± 1.41 | | |
|  |  | 6413 | *Fusarium* | 65.00 ± 1.60 | **76.00 ± 2.79** | 55.10 ± 4.98 | 30.02 ± 0.33 | 166,53 | 8.47 ± 0.22 | 0.00 ± 0.00 | 55.79 ± 0.01 | 17.69 ± 0.57 | 3.43 ± 0.58 | | |
|  |  | 6404 | *Neopestalotiopsis* | 50,03 ± 6,80 | 46,00 ± 1,90 | 48,73 ± 3,42 | 37,59 ± 7,98 | 133,02 | 1,07 ± 0,02 | 45,32 ± 6,48 | 0,00 ± 0,00 | 11,71 ± 0,71 | 0,67 ± 0,40 | | |
|  |  | 6406 | *Fusarium* | 33,30 ± 6,65 | 40,00 ± 0,00 | 33,30 ± 6,65 | 20,81 ± 8,47 | 240,26 | 12,60 ± 1,35 | 32,06 ± 2,40 | 17,32 ± 0,39 | 7,97 ± 0,19 | 4,38 ± 0,76 | | |
|  |  | 6446 | *Fusarium* | 50,34 ± 5,85 | 43,33 ± 11,79 | 38,72 ± 10,88 | 12,69 ± 10,29 | 394,16 | 9,66 ± 0,09 | 31,75 ± 0,18 | 14,99 ± 2,65 | 8,16 ± 0,08 | 32,76 ± 0,83 | | |
|  |  | 6463 | *Fusarium* | 61,29 ± 2,71 | 56,33 ± 6,06 | 48,78 ± 7,82 | 17,10 ± 12,82 | 292,35 | 23,49 ± 0,22 | 71,84 ± 1,07 | 37,03 ± 10,28 | 12,70 ± 0,12 | 6,16 ± 0,09 | | |
|  |  | 6425 | *Fusarium* | 58,00 ± 3,80 | 46,00 ± 3,65 | 55,95 ± 1,94 | 18,35 ± 7,80 | 272,55 | 3,11 ± 0,16 | 14,88 ± 0,45 | 21,38 ± 3,77 | 27,45 ± 0,45 | 7,42 ± 0,48 | | |
|  |  | 6426 | *Pseudopestalotiopsis* | 32,38 ± 9,04 | 40,67 ± 3,65 | 26,08 ± 3,88 | 37,59 ± 7,59 | 133,02 | 1,49 ± 0,03 | 12,01 ± 3,49 | 22,15 ± 3,99 | 12,65 ± 0,12 | 3,22 ± 0,32 | | |
|  |  | 6460 | *Fusarium* | 31,57 ± 14,10 | 30,67 ± 4,18 | 21,68 ± 8,79 | 29,29 ± 0,38 | 170,70 | 9,81 ± 0,16 | 0,00 ± 0,00 | 42,97 ± 9,11 | 6,68 ± 2,89 | 1,74 ± 1,42 | | |
|  |  | 6445 | *Fusarium* | 63.43 ± 2.10 | **69.00 ± 5.35** | 44.49 ± 14.38 | 9.51 ± 9.43 | 525,53 | 19.96 ± 0.37 | 32.18 ± 6.09 | 19.38 ± 0.07 | 14.07 ± 0.92 | 4.81 ± 0.40 | | |
|  |  | 6430 | *Fusarium* | 52.37 ± 5.80 | 44.00 ± 3.65 | 21.06 ± 6.86 | 37.59 ± 0.56 | **133,02** | **27.74 ± 0.34** | 40.20 ± 7.69 | 30.72 ± 7.00 | **41.33 ± 1.50** | | 11.29 ±0.77 |  |
|  | **Leaf** | 6439 | *Colletotrichum* | 56.43 ± 2.05 | 32.00 ± 1.83 | 32.46 ± 5.63 | 16.98 ± 9.42 | 294.52 | 4.68 ± 0.03 | 22.89 ± 7.20 | **155.79 ± 7.47** | 3.88 ± 0.20 | 6.06 ± 0.72 | | |
|  |  | 6443 | *Pithomyces* | 56.63 ± 8.09 | 44.67 ± 3.80 | 40.19 ± 4.45 | 4.85 ± 14,07 | 1029.87 | 8.47 ± 0.10 | 17.03 ± 0.45 | 15.53 ± 2.15 | 7.61 ± 0.35 | 29.82 ± 0.31 | | |
|  |  | 6419 | *Botryosphaeria* | 56.50 ± 7.47 | 30.67 ± 2.79 | 32.05 ± 5.25 | 19.24 ± 2.56 | 259.83 | 15.72 ± 0.62 | 50.84 ± 23.57 | 38.76 ± 10.89 | 40.16 ± 4.63 | 8.52 ± 0.09 | | |
|  |  | 6420 | *Diaporthe* | 63.40 ± 1.66 | 32.33 ± 4.01 | 37.23 ± 7.79 | ND | ND | 8.34 ± 1.92 | 14.10 ± 2.26 | 31.40 ± 9.83 | 21.32 ± 0.21 | 8.61 ± 2.67 | | |
|  |  | 6448 | *Pestalotiopsis* | 46.86 ± 2.94 | 34.33 ± 5.48 | 49.23 ± 1.06 | 29.57 ± 5.17 | 169.09 | 24.74 ± 0.85 | 27.98 ± 0,66 | 45.05 ± 12.88 | 24.91 ± 0.49 | 8.81 ± 0.65 | | |
|  |  | 6458 | *Fusarium* | 12.73 ± 7.15 | 37.33 ± 5.35 | 30.21 ± 2.40 | 29.57 ± 4.12 | 169.09 | 1.68 ± 0.34 | 12.33 ± 0.05 | 44.30 ± 13.99 | 16.95 ± 1.63 | 4.29 ± 0.29 | | |
|  |  | 6456 | *Fusarium* | 11.99 ± 2.25 | 18.33 ± 1.67 | 6.95 ± 3.88 | 4.85 ± 14.07 | 1029.87 | 9.15 ± 0.49 | 13.46 ± 3.34 | **108.86 ± 94.86** | 6.21 ± 0.32 | 12.90 ± 0.03 | | |
| ***Stryrax benzoin*** | **Bark** | 6398 | *Pestalotiopsis* | 61.10 ± 3.16 | 39.33 ± 2.79 | 37.95 ± 2.57 | 27.21 ± 0.98 | 183.72 | 10.06 ± 0.07 | 8.24 ± 3.71 | 38.75 ± 0.15 | 13,28 ± 0,00 | 2,07 ± 0,72 | | |
|  |  | 6409 | *Botryosphaeria* | 59.19 ± 7.26 | 26.67 ± 0.11 | 45.91 ± 6.91 | 29.29 ± 0.38 | 170.70 | 10.01 ± 0.04 | 46.67 ± 0.05 | 47.75 ± 10.12 | 14,11 ± 0,82 | 32,76 ± 0,83 | | |
|  |  | 6436 | *Neopestalotiopsis* | 69.40 ± 1.61 | 50.67 ± 1.90 | 42.37 ± 3.66 | 28.46 ± 0.39 | 175.68 | 5.00 ± 0.17 | 3.30 ± 4.93 | 6.27 ± 8.52 | 8,53 ± 0,65 | 1,25 ± 0,25 | | |
|  |  | 6449 | *Pestalotiopsis* | 14.69 ± 2.42 | 23.67 ± 4.15 | 6.31 ± 2.87 | 29.57 ± 2.65 | 169.09 | 1.68 ± 0.05 | 94.59 ± 24.17 | 67.52 ± 7.66 | 13,23 ± 0,32 | 6,49 ± 0,49 | | |
|  |  | 6464 | *Acremonium* | 15.11 ± 7.53 | 24.00 ± 3.84 | 30.56 ± 10.04 | 26.14 ± 1.72 | 191.30 | 9.24 ± 0.18 | 1.31 ± 3.04 | 27.72 ± 8.39 | 6,07 ± 0,54 | 2,01 ± 0,40 | | |
|  | **Stem** | 6399 | *Neofusicoccum* | 56.99 ± 18.96 | 59.00 ± 4.01 | **62.66 ± 3.25** | ND | ND | 22.21 ± 0.89 | **152.05 ± 32.40** | 29.07 ± 5.23 | 19.44 ± 1.05 | 1.34 ± 1.00 | | |
|  |  | 6431 | *Neopestalotiopsis* | 67.83 ± 2.52 | 46.33 ± 3.42 | 42.93 ± 4.36 | 49.65 ± 0.80 | **100.71** | 3.35 ± 0.14 | **197.49 ± 8.65** | 33.52 ± 0.28 | **43.02 ± 1.25** | **52.88 ± 4.93** | | |
|  |  | 6400 | *Pestalotiopsis* | 43.17 ± 3.08 | 30.00 ± 3.33 | 29.71 ± 15.71 | 15.91 ± 0.87 | 314.31 | 18.00 ± 0.06 | 116.15 ± 0.69 | 26.00 ± 1.98 | 13.61 ± 0.36 | 3.43 ± 1.59 | | |
|  |  | 6434 | *Neopestalotiopsis* | 37.64 ± 7.13 | 35.00 ± 3.33 | 11.06 ± 11.79 | 29.29 ± 0.38 | 170.70 | 3.91 ± 0.09 | ND | 40.22 ± 4.93 | 6.07 ± 1.47 | 1.37 ± 0.19 | | |
|  |  | 6437 | *Pestalotiopsis* | 62.65 ± 2.02 | 64.00 ± 3.46 | 46.79 ± 3.89 | 18.89 ± 0.44 | 264.71 | 9.51 ± 0.36 | 0.49 ± 0,84 | 60.16 ± 12.75 | 15.79 ± 3.76 | 14.91 ± 2.63 | | |
|  |  | 6438 | *Pestalotiopsis microspora* | 48.81 ± 9.04 | 42.00 ± 1.83 | 18.06 ± 7.11 | 24.11 ± 13.35 | 207.35 | 4.87 ± 0.94 | 106.27 ± 4,46 | 26.93 ± 7.47 | 6.59 ± 0.66 | 3.45 ± 0.73 | | |
|  |  | 6451 | *Neopestalotiopsis* | 61.49 ± 6.33 | 34.00 ± 5.96 | 48.86 ± 3.86 | 18.89 ± 0.44 | 264.71 | 7.59 ± 0.24 | 0.64 ± 1,02 | 49.10 ± 6.51 | 8.69 ± 0.25 | 1.90 ± 2.46 | | |
|  |  | 6450 | *Neopestalotiopsis* | 51.80 ± 3.64 | 32.00 ± 6.67 | 40.22 ± 9.98 | 7.27 ± 6.98 | 687.41 | 18.71 ± 1.36 | **167.39 ± 8.57** | 24.96 ± 8.72 | 9.95 ± 0.70 | 4.73 ± 0.07 | | |
|  | **Leaf** | 6401 | *Diaporthe* | 61.97 ± 5.38 | 44.00 ± 4.35 | 43.76 ± 5.99 | 29.16 ± 0,38 | 171.45 | 10.01 ± 0.18 | 12.07 ± 6.42 | 42.45 ± 0.07 | 15.34 ± 0.09 | 2.50 ± 0.35 | | |
|  |  | 6402 | *Fusarium* | 66.67 ± 2.36 | 48.67 ± 2.98 | 43.57 ± 5.04 | 17.99 ± 0.44 | 277.86 | 9.96 ± 0.11 | 10.45 ± 2.09 | 39.58 ± 8.39 | 11.73 ± 0.66 | 3.94 ± 0.34 | | |
|  |  | 6440 | *Clonostachys* | 35.96 ± 3.84 | 64.00 ± 1.90 | 37.17 ± 3.27 | ND | ND | 3.05 ± 0.11 | 132.46 ± 0.09 | 20.87 ± 1.98 | 11.23 ± 0.89 | 2.90 ± 0.22 | | |
|  |  | 6432 | *Diaporthe* | 61.22 ± 1.39 | 53.00 ± 1.39 | 19.23 ± 8.52 | 17,99 ± 0.44 | 277.86 | 6.40 ± 0.26 | ND | 50.13 ± 8.17 | 6.98 ± 0.08 | 5.62 ± 1.65 | | |
|  |  | 6455 | *Fusarium* | 39.64 ± 7.39 | 32.00 ± 5.06 | 25.85 ± 13.21 | 28.87 ± 0.97 | 173.16 | 10.04 ± 0.09 | 23.72 ± 1,26 | 3398 ± 10.28 | 17.92 ± 0.90 | 1.12 ± 0.54 | | |
|  |  | 6462 | *Fusarium* | 38.34 ± 7.66 | 38.00 ± 3.61 | 23.11 ± 6.78 | 28.87 ± 0.97 | 173.16 | 9.07 ± 0.11 | 1.45 ± 5,26 | 31.67 ± 6.71 | 7.11 ± 0.96 | 2.12 ± 0.71 | | |
|  |  | 6453 | *Fusarium* | 51.17 ± 3.05 | 46.67 ± 3.33 | 33.39 ± 17.57 | 30.34 ± 0.96 | 164.79 | 9.65 ± 0.11 | 23.43 ± 9,22 | 42.99 ± 13.01 | 24.25 ± 0.15 | 0.34 ± 0.90 | | |
| Remarks: Values are presented as mean ± SDs; DPPH: 2,2-diphenyl-1-picrylhydrazyl assay; ABTS: 2,2 ʹ -azino-bis(3-ethylbenzothiazoline-6-sulphonic acid) assay; FRAP: ferric reducing antioxidant power assay; ND: not detected; QE: quercetin equivalent; GAE: gallic acid equivalent; TE: Trolox equivalent. The highest three values in the phytochemical assays are presented in bold font. | | | | | | | | | | | | | | | |
